# Oral arsenic and retinoic acid for high-risk acute promyelocytic leukemia

**DOI:** 10.1186/s13045-022-01368-3

**Published:** 2022-10-18

**Authors:** Ya-Fang Ma, Ying Lu, Qian Wu, Yin-Jun Lou, Min Yang, Jie-Yu Xu, Cai-Hong Sun, Li-Ping Mao, Gai-Xiang Xu, Li Li, Jian Huang, Huai-Yu Wang, Li-Jiang Lou, Hai-Tao Meng, Jie-Jing Qian, Wen-Juan Yu, Ju-Ying Wei, Zhen-Yu Li, Xue-Lu Zhu, Xiao-Yan Yan, Su-Ning Chen, Jie Jin, Hong-Hu Zhu

**Affiliations:** 1 Department of Hematology, The First Affiliated Hospital, College of Medicine, Zhejiang University School of Medicine, #79 Qingchun Rd, Hangzhou, 310003 Zhejiang People’s Republic of China; 2 Zhejiang Provincial Key Laboratory of Hematopoietic Malignancy, Zhejiang University, Hangzhou, Zhejiang People’s Republic of China; 3 Zhejiang University Cancer Center, Hangzhou, Zhejiang People’s Republic of China; 4 Department of Hematology, Affiliated People’s Hospital of Ningbo University, Ningbo, Zhejiang People’s Republic of China; 5 Department of Hematology, First Affiliated Hospital of Soochow University, National Clinical Research Center for Hematologic Diseases, Jiangsu Institute of Hematology, Institute of Blood and Marrow Transplantation, Collaborative Innovation Center of Hematology, Soochow University, Suzhou, 215000 Jiangsu People’s Republic of China; 6 Program in Clinical Medicine, School of Medicine of Zhejiang University, Hangzhou, Zhejiang People’s Republic of China; 7 Department of Hematology, Fourth Affiliated Hospital, Zhejiang University School of Medicine, Yiwu, Zhejiang People’s Republic of China; 8 Department of Hematology, The First Affiliated Hospital of Xi’an Jiaotong University, Xi’an, Shaanxi, People’s Republic of China; 9 The First Hospital of Ninghai County, Zhejiang, People’s Republic of China; 10 The Affiliated Hospital of Xuzhou Medical University, Xuzhou, Jiangsu People’s Republic of China; 11 Peking University Clinical Research Institute, Beijing, People’s Republic of China; 12 Liangzhu Laboratory, Zhejiang University Medical Center, Hangzhou, Zhejiang People’s Republic of China

**Keywords:** Phase 2 clinical trial, Oral arsenic, Realgar–Indigo naturalis formula, High-risk APL, Consolidation therapy

## Abstract

Acute promyelocytic leukemia (APL) has become curable over 95% patients under a complete chemo-free treatment with all-trans retinoic acid (ATRA) and arsenic trioxide in low-risk patients. Minimizing chemotherapy has proven feasible in high-risk patients. We evaluated oral arsenic and ATRA without chemotherapy as an outpatient consolidation therapy and no maintenance for high-risk APL. We conducted a multicenter, single-arm, phase 2 study with consolidation phases. The consolidation therapy included Realgar–Indigo naturalis formula (60 mg/kg daily in an oral divided dose) in a 4-week-on and 4-week-off regimen for 4 cycles and ATRA (25 mg/m^2^ daily in an oral divided dose) in a 2-week-on and 2-week-off regimen for 7 cycles. The primary end point was the disease-free survival (DFS). Secondary end points included measurable resident disease, overall survival (OS), and safety. A total of 54 participants were enrolled at seven centers from May 2019. The median age was 40 years. At the median follow-up of 13.8 months (through April 2022), estimated 2-year DFS and OS were 94% and 100% in an intention-to-treat analysis. All the patients achieved complete molecular remission at the end of consolidation phase. Two patients relapsed after consolidation with a cumulative incidence of relapse of 6.2%. The majority of adverse events were grade 1–2, and only three grade 3 adverse events were observed. Oral arsenic plus ATRA without chemotherapy was active as a first-line consolidation therapy for high-risk APL.

*Trial registration*: chictr.org.cn number, ChiCTR1900023309.

**Supplementary Information:**

The online version contains supplementary material available at 10.1186/s13045-022-01368-3.

**To the editor,**

Acute promyelocytic leukemia (APL) has now become a highly curable disease with all-trans retinoic acid (ATRA) and arsenic trioxide (ATO) combined treatment. [1, 2] For low-risk patients, defined by a presenting white blood cell (WBC) count ≤ 10 × 10^9^/L, a survival over 95% could be achieved using only ATRA and ATO without chemotherapy in most recent studies. [3–10]

For high-risk APL patients (WBC count > 10 × 10^9^/L), two randomized controlled trial (RCT) studies have shown that ATRA + ATO + gemtuzumab ozogamicin/anthracycline as consolidation treatment had a high cure rate and possibly lower relapse rate than ATRA + chemotherapy. [9, 11] One RCT is ongoing to compare ATRA plus ATO vs ATRA plus chemotherapy as consolidation treatment in Europe (registered at www.clinicaltrials.gov as #NCT02688140), and the results have not been reported yet. To further simply the post-remission treatment, we design a protocol using oral arsenic and ATRA without chemotherapy as a first-line consolidation therapy and no maintenance treatment. In our pilot study, the 3-year estimated overall survival (OS) and event-free survival were 100% and 89.4% in 20 high-risk APL patients. [12]

From May 2019, we conducted a multicenter, single-arm, phase 2 study to assess the efficacy and safety of Realgar–Indigo naturalis formula (RIF) plus ATRA as consolidation therapy under an outpatient model for high-risk APL. This trial is ongoing and is registered with Chinese Clinical Trial Registry, ChiCTR1900023309. The protocol and detailed scheme are shown in supplemental material (Protocol, Additional file 1: Figure S1).

Fifty-four eligible patients were enrolled, and an intention-to-treat (ITT) analysis was performed. Excluding 15 patients who did not complete the entire consolidation therapy, 39 patients were included in the per-protocol (PP) analysis (Additional file 1: Figure S2). The clinical characteristics of patients in ITT and PP analysis are shown in Additional file 1: Table S1. There were 28 males and 26 females, and the median age was 40 years old (17–77 years). The median PML-RARA at enrollment was 0.19% (0–99%). Thirteen patients (24%) received ATRA + ATO/RIF without chemotherapy, and 41 patients (76%) received ATRA + ATO/RIF + chemotherapy as induction regimen. Chemotherapy drugs included anthracycline, cytarabine, and homoharringtonine. RIF and ATO were used in 42 (77.8%) and 12 (22.2%) patients in induction therapy, respectively. Five patients had cerebral hemorrhage in induction therapy, and one of them had brain surgery (Additional file 1: Table S2).

With a median follow-up of 13.8 months, the primary end point has been met as estimated 2-year disease-free survival (DFS) was 93.8% (95% CI: 76.9–98.4%) in ITT analysis. The estimated 2-year OS was 100% in ITT analysis. Estimated 2-year DFS was 93.1% (95% CI: 74.8–98.3%), and estimated 2-year OS was 100% in PP analysis (Fig. 1). These were consistent with the data from clinical trials which just use ATRA and ATO as consolidation therapy, with a DFS of 89% (5 years, *n* = 52), [3] 89.4% (3 years, *n* = 20), [12] and 96.4% (2 years, *n* = 56, Pediatric), [10], respectively. In addition, the survival was also comparable with the data from clinical trials which use ATRA + ATO + chemotherapy as consolidation therapy, with a DFS of 92% (2 years, *n* = 20), [7] 90.4% (3 years, *n* = 235), [11].

**Fig. 1:**
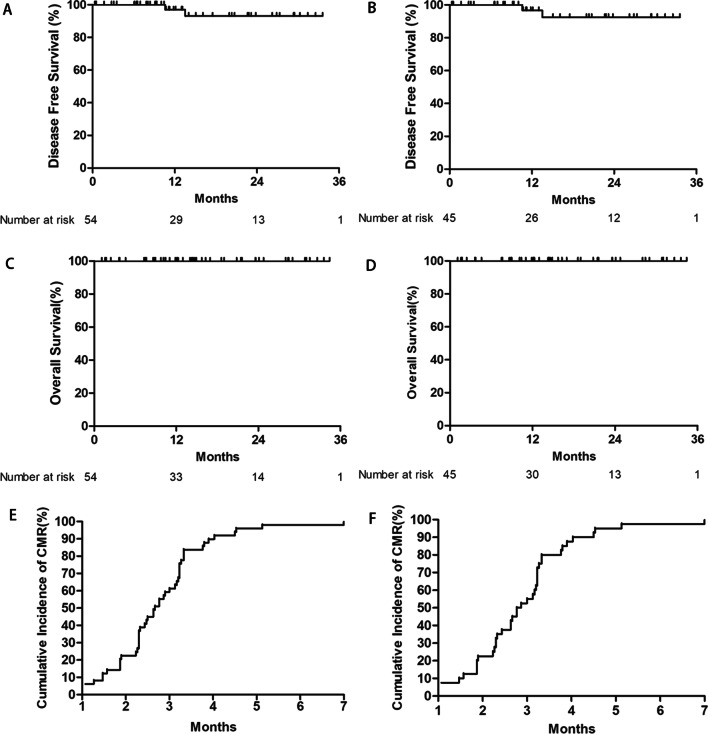
Survival among patients in ITT analysis and PP analysis. **A** DFS in ITT analysis; **B** DFS in PP analysis; **C** OS in ITT analysis; **D** OS in PP analysis; **E** Cumulative incidence of CMR from the beginning of the consolidation therapy in ITT analysis; and **F** Cumulative incidence of CMR in PP analysis. The ITT population comprised patients who enrolled and received at least one dose of study medication. The PP population comprised patients of a subgroup of ITT population who had completed the trial and underwent an end-of-therapy evaluation. Abbreviations: ITT: intention-to-treat; PP: per-protocol; DFS: disease-free survival; OS: overall survival; and CMR: complete molecular remission

Except for 3 patients who were receiving consolidation therapy, the remaining 51 patients all achieved complete molecular remission (CMR), and nearly 90% of them achieved CMR at 4 months from the beginning of induction therapy (Fig. 1). The results of PML-RARA fusion gene test are shown in Additional file 1: Figure S3. Totally, only two patients occurred relapses with a cumulative incidence of relapse at 2 years of 6.2%. Detailed information of these two patients is shown in Additional file 1: Table S3.

Of the 52 participants who completed at least one cycle of consolidation therapy, 48 completed an adverse event survey. No grade 4–5 adverse events were observed. The most common non-hemopoietic toxic effects were headaches (15%), arthralgia and myalgia (19%), dry (19%), darkened skin (10%), and alopecia (10%). Adverse events such as infection, renal dysfunction, and cardiac toxicity were not observed. The majority of adverse events were grade 1–2, and only three grade 3 adverse events were observed. No patient died in the consolidation phase (Table 1).

**Table 1:** Adverse events

Adverse event	Grade 1	Grade 2	Grade3	Grade4	Grade5
Gastrointestinal reaction					
Nausea and vomiting	3 (6%)	0	1 (2%)	0	0
Diarrhea	2 (4%)	2 (4%)	0	0	0
Hematochezia	0	1 (2%)	2 (4%)	0	0
Pain					
Abdominal pain	1 (2%)	0	0	0	0
Headache	6 (13%)	1 (2%)	0	0	0
Arthralgia and Myalgia	7 (15%)	2 (4%)	0	0	0
Chest pain	2 (4%)	0	0	0	0
Skin and mucosa					
Dry	9 (19%)	0	0	0	0
Darkened skin	5 (10%)	0	0	0	0
Alopecia	5 (10%)	0	0	0	0
Rash	1 (2%)	3 (6%)	0	0	0
Other symptoms					
Fatigue	3 (6%)	1 (2%)	0	0	0
Tinnitus	1 (2%)	0	0	0	0
Epistaxis	2 (4%)	0	0	0	0
Laboratory test					
Leukocytopenia	1 (2%)	1 (2%)	0	0	0
Thrombocytopenia	1 (2%)	0	0	0	0
Hepatic dysfunction	1 (2%)	0	0	0	0

Data are *n* (%)

Three grade 3 adverse events were observed. One patient stopped treatment for 2 months due to grade 3 gastrointestinal reaction and then returned to regular treatment. The other one had abdominal pain and hematochezia at diagnosis, and grade 3 hematochezia occurred again during consolidation therapy. Her enteroscopy suggested ischemic bowel disease. Her consolidation therapy was subsequently changed to chemotherapy and followed by ATRA for maintenance therapy. The last one also had grade 3 hematochezia in the third cycle of consolidation therapy. His abdominal CT scan and enteroscopy suggested intussusception and ischemic bowel disease

In summary, this trial found that adult patients with high-risk APL could be successfully and safely treated with oral arsenic plus ATRA as consolidation therapy. A completely oral and chemo-free post-remission treatment without maintenance under an outpatient model will benefit more APL patients.

## Supplementary Information

**Additional file 1. Figure S1.** The treatment schema. The consolidation therapy included RIF (60 mg/kg daily in an oral divided dose) in a 4-week-on and 4-week-off regimen for 4 cycles and ATRA (25 mg/m2 daily in an oral divided dose) in a 2-week-on and 2-week-off regimen for 7 cycles. Of note, treatment cycles were counted according to the cycles of ATRA. **Figure S2.** Flow chart of participants enrollment, treatment and follow-up. 54 patients were enrolled in the clinical trial and an intention-to-treat analysis were performed. Seven patients had major protocol violation. Four of them suffered cerebral hemorrhage in induction therapy, so their physicians advised two cycles of chemotherapy for consolidation therapy and continued with RIF plus ATRA for 7 cycles. One of them changed protocol for a grade 3 hematochezia. One patient returned to his hometown hospital and changed his treatment plan. The last one did not give a specific reason for the change in treatment. Another seven patients were in consolidation process. Two patients were lost to follow-up and one of them had not completed the trial. Thus 39 patients were included in the per-protocol analysis. **Figure S3.** PML-RARA tested at 3, 5 and 7 months from the beginning of the induction therapy in ITT analysis.**Table S1.** Characteristics of the Patients. **Table S2.** Clinical characteristics and treatment of five patients with cerebral hemorrhage. **Table S3.** Treatment and outcomes of two relapsed patients. Patient No.1 had cerebral hemorrhage at the time of diagnosis. She had a molecular relapse at 11 months after remission and a central nervous system relapse at 13 months. Patient No.2 had a hematologic relapse at 13 months and achieved remission again after RIF plus ATRA reinduction and alive until last follow-up.

## Data Availability

The datasets used and/or analyzed during the current study are available from the corresponding author on reasonable request.

## Abbreviations

APL: Acute promyelocytic leukemia
ATRA: All-trans retinoic acid
ATO: Arsenic trioxide
WBC: White blood cell
RCT: Randomized controlled trial
OS: Overall survival
RIF: Realgar–Indigo naturalis formula
ITT: Intention-to-treat
PP: Per-protocol
DFS: Disease-free survival
CMR: Complete molecular remission
