# Supplementary material for: Oral arsenic and retinoic acid for high-risk acute promyelocytic leukemia
Source: J Hematol Oncol. 2022 Oct 18;15:148. doi: 10.1186/s13045-022-01368-3 (PMC9578225; doi:10.1186/s13045-022-01368-3)
Supplement: Supplementary file 1 — Additional file 1. Figure S1. The treatment schema. The consolidation therapy included RIF (60 mg/kg daily in an oral divided dose) in a 4-week-on and 4-week-off regimen for 4 cycles and ATRA (25 mg/m2 daily in an oral divided dose) in a 2-week-on and 2-week-off regimen for 7 cycles. Of note, treatment cycles were counted according to the cycles of ATRA. Figure S2. Flow chart of participants enrollment, treatment and follow-up. 54 patients were enrolled in the clinical trial and an intention-to-treat analysis were performed. Seven patients had major protocol violation. Four of them suffered cerebral hemorrhage in induction therapy, so their physicians advised two cycles of chemotherapy for consolidation therapy and continued with RIF plus ATRA for 7 cycles. One of them changed protocol for a grade 3 hematochezia. One patient returned to his hometown hospital and changed his treatment plan. The last one did not give a specific reason for the change in treatment. Another seven patients were in consolidation process. Two patients were lost to follow-up and one of them had not completed the trial. Thus 39 patients were included in the per-protocol analysis. Figure S3. PML-RARA tested at 3, 5 and 7 months from the beginning of the induction therapy in ITT analysis.Table S1. Characteristics of the Patients. Table S2. Clinical characteristics and treatment of five patients with cerebral hemorrhage. Table S3. Treatment and outcomes of two relapsed patients. Patient No.1 had cerebral hemorrhage at the time of diagnosis. She had a molecular relapse at 11 months after remission and a central nervous system relapse at 13 months. Patient No.2 had a hematologic relapse at 13 months and achieved remission again after RIF plus ATRA reinduction and alive until last follow-up. [file 13045_2022_1368_MOESM1_ESM.docx]

Supplementary Appendix

Figure S1. The treatment schema. The consolidation therapy included RIF (60 mg/kg daily in an oral divided dose) in a 4-week-on and 4-week-off regimen for 4 cycles and ATRA (25 mg/m2 daily in an oral divided dose) in a 2-week-on and 2-week-off regimen for 7 cycles. Of note, treatment cycles were counted according to the cycles of ATRA.


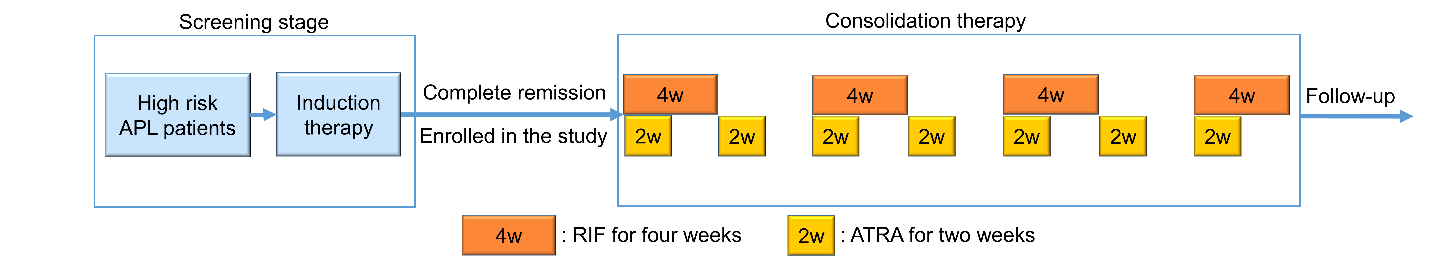


Figure S2. Flow chart of participants enrollment, treatment and follow-up. 54 patients were enrolled in the clinical trial and an intention-to-treat analysis were performed. Seven patients had major protocol violation. Four of them suffered cerebral hemorrhage in induction therapy, so their physicians advised two cycles of chemotherapy for consolidation therapy and continued with RIF plus ATRA for 7 cycles. One of them changed protocol for a grade 3 hematochezia. One patient returned to his hometown hospital and changed his treatment plan. The last one did not give a specific reason for the change in treatment. Another seven patients were in consolidation process. Two patients were lost to follow-up and one of them had not completed the trial. Thus 39 patients were included in the per-protocol analysis.


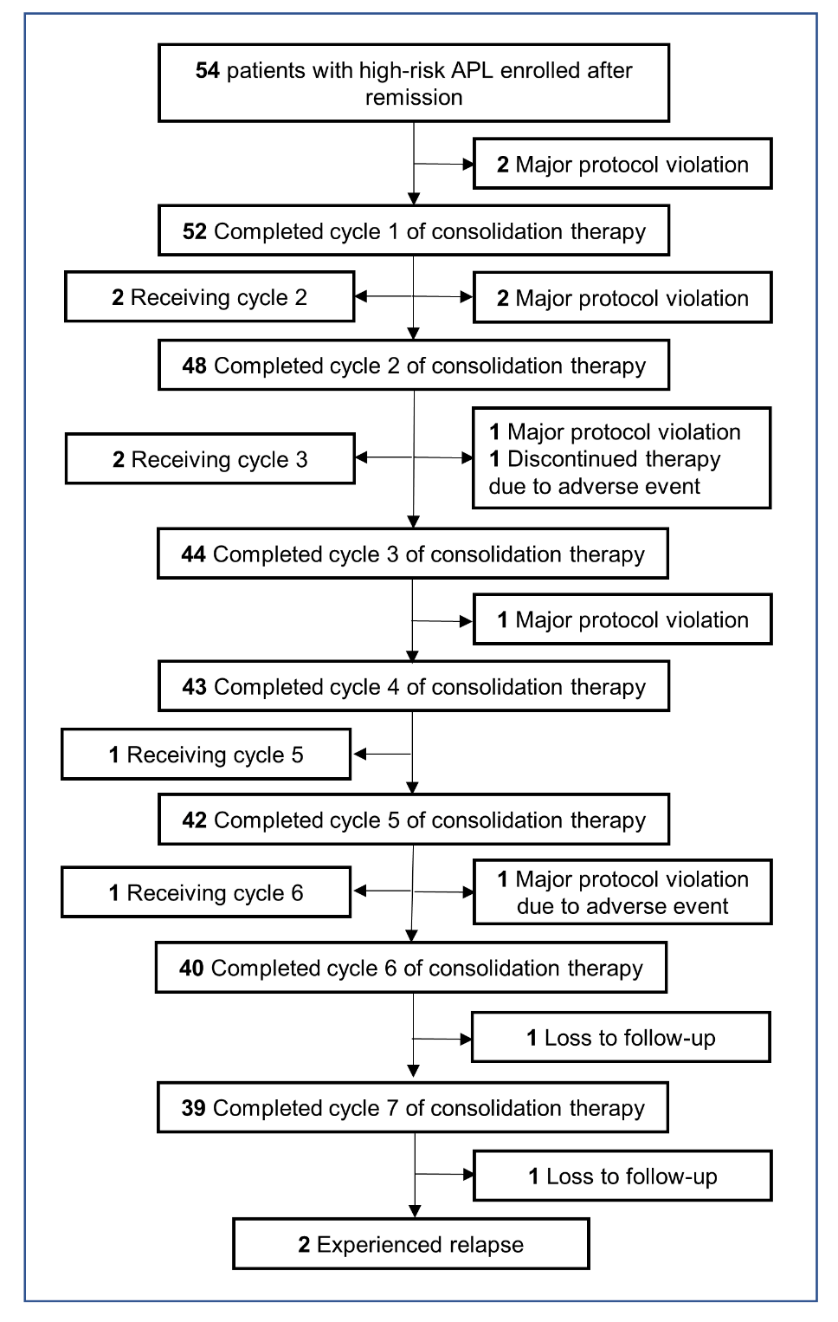


Figure S3. PML-RARA tested at 3, 5 and 7 months from the beginning of the induction therapy in ITT analysis.


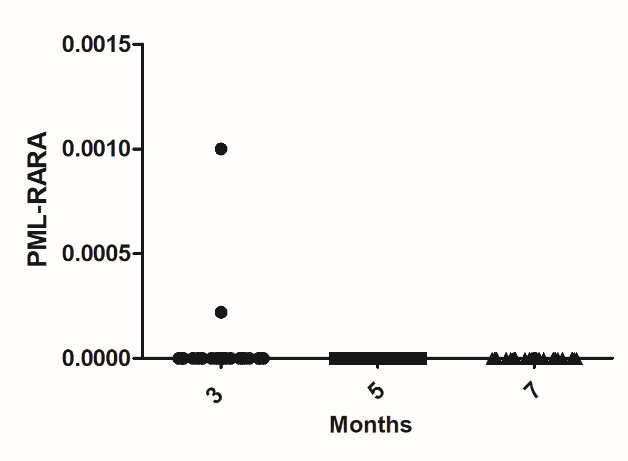


Table S1：Characteristics of the Patients

| Characteristics | ITT (n=54) | PP (n=39) |
| --- | --- | --- |
| Age (years)  Median (range) | 40(27-54; 17-77) | 33(27-52; 17-77) |
| 16-19 | 4(7%) | 4(10%) |
| 20-39 | 22(41%) | 17(44%) |
| 40-59 | 25(46%) | 15(38%) |
| ≥60 | 3(6%) | 3(8%) |
| Sex  Male  Female | 28(52%)  26(48%) | 17(44%)  22(56%) |
| Laboratory data at the onset |  |  |
| PML-RARA  Median (range) | 0.67(0.38-0.94; 0.12-1.00) | 0.66(0.37-0.86; 0.12-1.00) |
| WBC count (10^9^/L)  Median (range) | 27.3(17.0-56.1; 10.2-158.7) | 30.1(17.5-57.7; 10.2-158.7) |
| (10-20) ×10^9^/L | 20(37%) | 14(36%) |
| (21-50) ×10^9^/L | 18(33%) | 13(33%) |
| >50×10^9^/L | 16(30%) | 12(31%) |
| Platelet count (10^9^/L)  Median (range) | 26 (13-44; 4-91) | 33 (16-46; 4-91) |
| Fibrinogen (g/L)  Median (range) | 1.26 (0.76-1.70; 0.43-3.74) | 1.33(0.80-2.00; 0.43-3.74) |
| Blasts in BM (%)  Median (range) | 88 (83-92; 43-99) | 88 (80-92; 43-97) |
| Additional cytogenetic abnormalities | 3(6%) | 1(3%) |
| Cerebral hemorrhage in induction therapy | 5(9%) | 1(3%) |
| RIF in induction therapy | 42(78%) | 31(79%) |
| PML-RARA at enrollment  Median (range) | 0.0019(0.0002-0.1072; 0-0.99) | 0.0015(0.0002-0.1015; 0-0.99) |
| CMR at enrollment | 7(13%) | 4(10%) |

Data are median (IQR; range) or n (%); ITT: intention-to-treat; PP: per-protocol; WBC: white blood cell; BM: bone marrow; RIF: realgar indigo formula.

Table S2. Clinical characteristics and treatment of five patients with cerebral hemorrhage.

| Num | Sex | Age  (years) | WBC (10^9^/L) | PLT (10^9^/L) | Fib (g/L) | D-dimer (ug/L） | Gene mutation | Brain surgery | Induction therapy | Post-remission therapy | Time to CMR(days) | CNS prophylaxis | Outcome |
| --- | --- | --- | --- | --- | --- | --- | --- | --- | --- | --- | --- | --- | --- |
| 1 | F | 55 | 23.67 | 37 | 0.81 | 7268 | ND | No | ATRA+ATO/RIF | ATRA+RIF | 69 | Yes | Relapse |
| 2 | M | 51 | 14.63 | 16 | 0.74 | 24414 | FLT3-ITD  FLT3-TKD | No | ATRA+ATO/RIF | ATRA+RIF+medium dose of cytarabine×2 cycles | 87 | Yes | CMR |
| 3 | M | 40 | 59.2 | 19 | 0.56 | 38507 | ND | No | ATRA+IDA+ATO/RIF | ATRA+RIF+medium dose of cytarabine×2 cycles | 74 | Yes | CMR |
| 4 | F | 41 | 96.02 | 8 | 1.53 | >20000 | ND | No | ATRA+IDA+ATO | ATRA+RIF+medium dose of cytarabine×2 cycles | 44 | Yes | CMR |
| 5 | M | 21 | 23.2 | 11 | 1.49 | 75000 | FLT3-ITD  NPM1 | Yes | ATRA+IDA+ATO | ATRA+RIF+medium dose of cytarabine×2 cycles | 100 | Yes | CMR |

ND: not done; WBC: white blood cell; PLT: platelet; ATRA: all-trans retinoic acid; ATO: arsenic trioxide; RIF: realgar-indigo naturalis formula;

IDA: Idarubicin; CMR: complete molecular remission; CNS: central nervous system.

Table S3. Treatment and outcomes of two relapsed patients. Patient No.1 had cerebral hemorrhage at the time of diagnosis. She had a molecular relapse at 11 months after remission and a central nervous system relapse at 13 months. Patient No.2 had a hematologic relapse at 13 months and achieved remission again after RIF plus ATRA reinduction and alive until last follow-up.

| Num | Sex | Age  (years) | Induction therapy | Time to CMR  (days) | Time to relapse | Treatment after relapse | Outcome |
| --- | --- | --- | --- | --- | --- | --- | --- |
| 1 | M | 49 | ATRA+ATO | 80 | Hematologic relapse at 13 months after remission | RIF+ATRA | Complete remission again;  Alive now |
| 2 | F | 55 | ATRA+ATO/RIF | 69 | Molecular relapse at 11 months after remission;  Central relapse at 13 months after remission | Medium dose of cytarabine×1 cycle;  Venetoclax+CAG regimen×1 cycle | Have not achieve remission;  Alive now |

ATRA: all-trans retinoic acid; ATO: arsenic trioxide; RIF: realgar-indigo naturalis formula; CMR: complete molecular remission; CAG: cytarabine 10mg/m^2^ q12h day1-14+ aclacinomycin 20mg day1-4+ Granulocyte-stimulating factor
